# Placental pathology and maternal factors associated with stillbirth: An institutional based case-control study in Northern Tanzania

**DOI:** 10.1371/journal.pone.0243455

**Published:** 2020-12-31

**Authors:** Godwin Lema, Alex Mremi, Patrick Amsi, Jeremia J. Pyuza, Julius P. Alloyce, Bariki Mchome, Pendo Mlay

**Affiliations:** 1 Department of Obstetrics and Gynecology, Kilimanjaro Christian Medical University College, Moshi, Tanzania; 2 Department of Pathology, Kilimanjaro Christian Medical Center, Moshi, Tanzania; 3 Cancer Registry Unit, Cancer Care Centre, Kilimanjaro Christian Medical Centre, Moshi, Tanzania; 4 Department of Obstetrics and Gynecology, Kilimanjaro Christian Medical Centre, Moshi, Tanzania; University of Mississippi Medical Center, UNITED STATES

## Abstract

**Objective:**

To determine the placental pathologies and maternal factors associated with stillbirth at Kilimanjaro Christian Medical Centre, a tertiary referral hospital in Northern Tanzania.

**Methods:**

A 1:2 unmatched case-control study was carried out among deliveries over an 8-month period. Stillbirths were a case group and live births were the control group. Respective placentas of the newborns from both groups were histopathologically analyzed. Maternal information was collected via chart review. Mean and standard deviation were used to summarize the numerical variables while frequency and percentage were used to summarize categorical variables. Crude and adjusted logistic regressions were done to test the association between each variable and the risk of stillbirth.

**Results:**

A total of 2305 women delivered during the study period. Their mean age was 30 ± 5.9 years. Of all deliveries, 2207 (95.8%) were live births while 98 (4.2%) were stillbirths. Of these, 96 stillbirths (cases) and 192 live births (controls) were enrolled. The average gestational age for the enrolled cases was 33.8 ±3.2 weeks while that of the controls was 36.3±3.6 weeks, (*p-value* 0.244). Of all stillbirths, nearly two thirds 61(63.5%) were males while the females were 35(36.5%). Of the stillbirth, 41were fresh stillbirths while 55 were macerated. The risk of stillbirth was significantly associated with lower maternal education [aOR (95% CI): 5.22(2.01–13.58)], history of stillbirth [aOR (95%CI): 3.17(1.20–8.36)], lower number of antenatal visits [aOR (95%CI): 6.68(2.71–16.48), pre/eclampsia [aOR (95%CI): 4.06(2.03–8.13)], and ante partum haemorrhage [OR (95%CI): 2.39(1.04–5.53)]. Placental pathology associated with stillbirth included utero-placental vascular pathology and acute chorioamnionitis.

**Conclusions:**

Educating the mothers on the importance of regular antenatal clinic attendance, monitoring and managing maternal conditions during antenatal periods should be emphasized. Placentas from stillbirths should be histo-pathologically evaluated to better understand the possible aetiology of stillbirths.

## Introduction

Stillbirth refers to a baby born at or beyond 28 weeks of gestation or if weight is >1000gram when gestation age is not known with no sign of life [1]. An estimated 2.6 million stillbirths are recorded worldwide yearly. Approximately 98% of stillbirths occur in low and middle-income countries (LMICs), of which over two-thirds were in sub-Saharan Africa (SSA) and Southern Asia [1]. The stillbirth rate in SSA is approximately 10 times that of high-income countries (29 vs. 3 per 1000 births) [1]. According to the WHO the stillbirth rate (SBR) in SSA ranges from as high as in 42 per 1000 live births in Nigeria and as low as 22 per 1000 live births in Kenya [2, 3]. The highest SBR observed in the East Africa is in Tanzania, noted to be 25.9 per 1000 live births [1].

Several interventions have been done to reduce the rates of stillbirth worldwide including, the global Every New-born Action Plan (ENAP) in 2014. It set a target for national SBRs of 12 or fewer stillbirths per 1000 births in all countries by 2030, accompanied by plans for countries to address disparities [4]. A huge effort is still needed by most African countries to achieve the ENAP target by the year 2030. The recommendation of placenta histopathology after stillbirth is strongly supported by individual reports showing placenta abnormalities are contributory in over 60% of stillbirths [5, 6]. The essential role of the placenta in maintaining a healthy pregnancy and evidence of placental pathology in several clinical conditions associated with increased SBR make placental histopathology an essential aspect of the epidemiology of stillbirth [7, 8].

The discrepancy in the incidence rate of stillbirths between high-income countries and sub-Sahara Africa show that most stillbirths can be prevented [9]. Data describing placenta abnormalities among stillbirth cases are virtually non-existent in SSA countries including Tanzania. We conceived the present study to assess the commonest placental pathologies as well as the maternal risk factors associated with stillbirth in order to better understand the possible aetiologies of the stillbirth which is essential for preventative measures.

## Materials and methods

### Study design, area, and period

This was the unmatched hospital-based case control-study conducted among deliveries at Kilimanjaro Christian Medical Centre (KCMC), from October 2018 to May 2019. The study was conducted in the obstetric ward of the Department of Obstetrics and Gynaecology as well as Department of Pathology. KCMC is a tertiary referral hospital that receives obstetrical patients from a local community in the Kilimanjaro region as well as from nearby regions in Northern Tanzania. The hospital has an annual delivery volume of about 4000 total births. It’s one among the major three zonal referral consultant and University Teaching Hospitals in Tanzania which was established in 1971. According to the national population census 2011/2012 report, Kilimanjaro region had a total population of 1,640,087 [10].

### Eligibility criteria

#### Inclusion criteria and case definition

The study included pregnant women with gestation age of 28 weeks and above who delivered at KCMC, and who consented to participate in the study. A case was any mother that delivered a stillbirth infant. The controls were mothers that delivered a live born infant during the study period, and each recruited case, two controls were selected by using the first and third delivery after a case within 24 hours period.

#### Exclusion criteria

Infants with obvious congenital malformations were all excluded from the study.

#### Sample size and data collection

Sample size was calculated using Fleiss formula online calculator. Using a 95% confidence interval, a minimum power to detect a difference of 80%, the hypothetical ratio of control to cases of 2:1 and assuming a minimum odd ratio of 2 for differences to be detected based on previous studies with similar design [11]. We estimated the minimum sample size at 288, consisting of 96 cases and 192 controls.

The main dependent variable of interest was stillbirth; while independent variables included maternal demographic characteristics, obstetrical history, medical conditions and placenta pathologies. Of maternal demographic characteristics; age, education level, marital status and alcohol consumption were matched between the cases and control while history of stillbirth, parity, antenatal care visits, pre/eclampsia and preterm premature rupture of membrane (PPROM) were obstetrical conditions that were matched between the cases and control. Of medical conditions, Diabetes in pregnancy, anaemia, HIV and malaria were matched between the cases and control. Of placenta pathologies; nature of inflammatory reaction, uteroplacental vascular pathology, and coagulation related lesions, umbilical cord findings including oedema, thrombosis, hematoma, necrosis, knots, and torsion were matched among cases and control groups.

A structured questionnaire was employed for data collection (S1 Questionnaire). The questionnaire was comprised of 29 questions that included socio-demographic data (residence, education, occupation, marital status, and age) as well as the medical, surgical, and obstetrical history of the mothers. Initial weight and height were extracted from the patient’s antenatal card. Placental pathology reports were obtained from the Pathology Department of KCMC. The principal investigator and /or an assistant approached the potential participants in the obstetrical ward and screened them for eligibility into the study. Briefly, all pregnant women with gestation age of 28 weeks and above who attended KCMC during the defined study time frame (from October 2018 to May 2019,) were approached by the study team before delivery and introduced about study objectives. Only those who met inclusion criteria and voluntarily gave an informed consent to participate in this study were enrolled. As the study had two different groups (cases and controls), the consent process prior to delivery was primarily intended to obtain the controls. However, despite obtaining authorization to use placenta for the study from all consented women; the number of controls enrolled and thus included in the analysis was determined by the availability of cases at the ratio of 1:2 between the cases and controls respectively.

Women who happened to have intrapartum stillbirths (fresh stillbirths), were re-approached by our study team immediately post delivery and asked for the second consent to be included in the cases category since initially were recruited as controls. Their respective placentas were already preserved in formalin as control during re-consenting process. The remaining placentas were disposed according to the hospital protocol in line with the Tanzanian Ministry of Health, Community Development, Gender, Elderly and Children, [12]. Expected cases prior to delivery were pregnant women whom during clinical presentation and investigations were confirmed to be having intrauterine foetal demise and thus macerated stillbirths were expected. After an explanation of the study objectives and obtained an informed consent, the patient was interviewed while completing the questionnaire. Additional information was obtained from the medical record and antenatal card if available.

Immediately after delivery, the placentas together with umbilical cords and membranes from the women who were enrolled into the study were grossly examined and kept in a well-labelled clean container with a 10% buffered formalin solution and then submitted to the Department of Pathology for both macroscopic and microscopic examinations. An experienced laboratory scientist processed the specimens, prepared microscopic glass slides and blinded the pathologists about the category as to whether the slides were from the cases or the control group. The slides were reviewed by the two study pathologists independently. Tele-pathology consultation was sought from sub-specialized gynaecological-pathologists at DUKE University or University of California San Francisco (USA) if there was a discrepancy in the diagnoses between the two study pathologists. The placenta histological lesions were classified based on Salafia method [7]. Briefly, the histological lesions were classified into the following groups; 1) Acute chorioamnionitis, 2) Chronic inflammation in the presence of features of villitis, 3) Uteroplacental vascular pathology features and evidence of secondary villous damage in the presence of severe villous fibrosis, villous infarct, fibrinoid necrosis, and vascular hypertrophy, and 4) Coagulation related lesions in the presence of uteroplacental vascular thrombosis, hemorrhagic endovasculitis and perivillous fibrin deposits. Cases were attributed to placenta or umbilical factors, if histopathology showed greater than 40% infarction or extensive involvement of the placenta with vascular or inflammatory changes. If there are no significant pathologic findings was classified as a normal placenta [7].

#### Data analysis

Data were analysed using STATA 13.0. Data quality was checked for completeness, timeliness and validity of the pathological findings. Numerical data were summarized using mean and median with their respective measure of spread while categorical data were summarized using frequencies and percentages. Chi-square or Fisher's exact tests were used to measure the relationship between risk factors in-relation to occurrence of a stillbirth, these were considered statistically significant if they have a p-value of less than or equal to 0.05. Independent risk factors were run using bivariate logistic regression for crude odds ratios to determine the association of each and the occurrence of stillbirth. Statistically significant factors in crude odds ratio were further adjusted to control possible intonations ‘confounders and modified effect’ using multivariate logistic regression. A 95% CI that does not include 1 was regarded as a significant risk factor.

Ethical clearance for this study was obtained from Kilimanjaro Christian Medical College Research Ethics and Review Committee with clearance number 2357. For all participants aged 18 years and above, a written informed consent to participate in this study was sought and obtained. Participants who were under 18 years old, a written informed consent was obtained either from their respective spouses, parents or legal guardians by signing the consent forms after risk and benefit of participating into the study were described. Participation was absolutely voluntary and the entire research was carried in accordance with Helsinki declaration.

## Results

**Fig 1:**
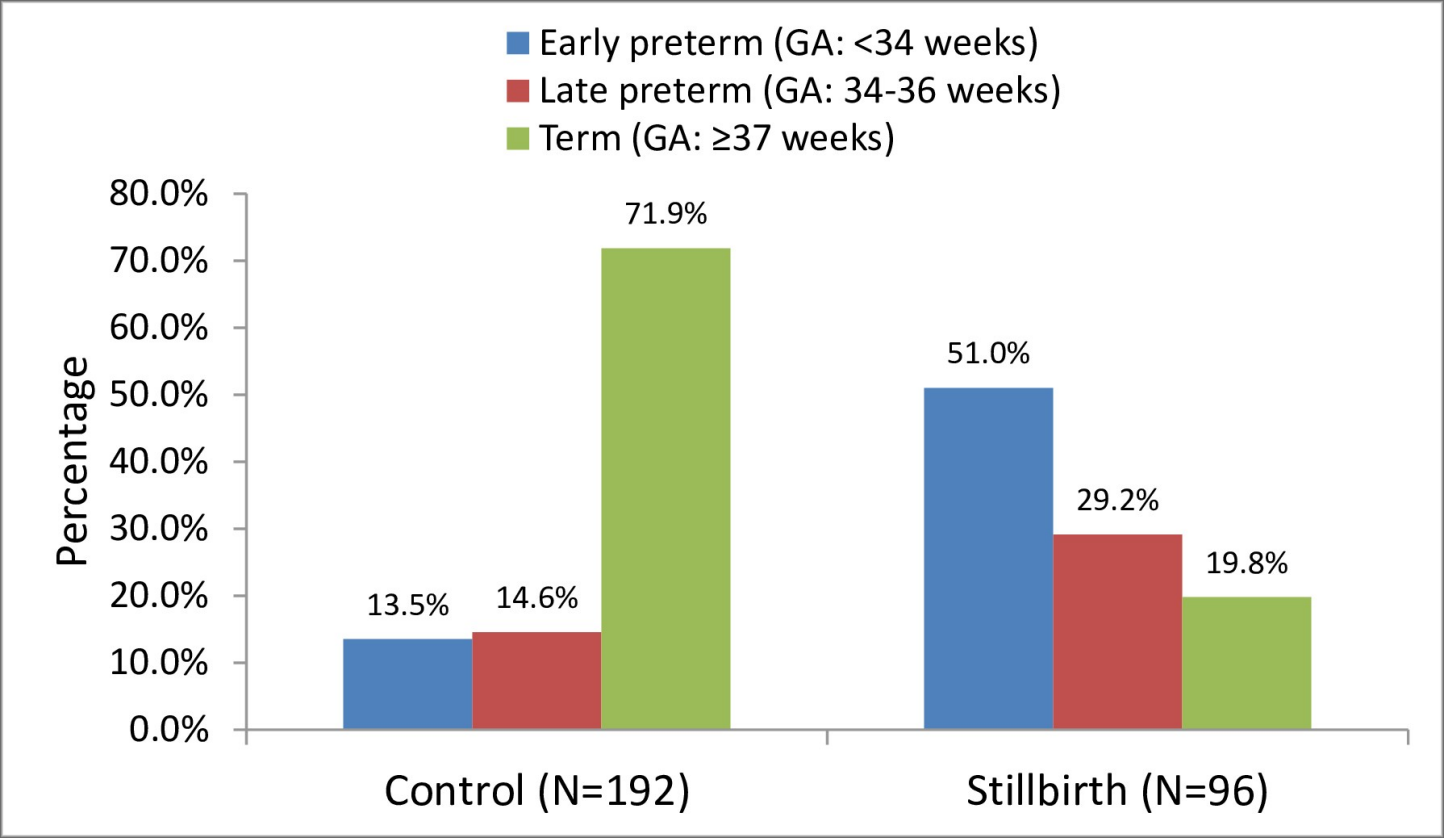
Distribution of the cases and controls by gestational age, (n = 288).

A total of 2305 women delivered from October 2018 to May 2019. Of these, 98 (4.2%) were stillbirths and 2207 were live births (95.8%). A total of 288 deliveries were recruited into the study which included 96 stillbirths (cases) and 192 live births (controls). Two stillbirths were excluded because of the gross congenital malformations. Distribution of the cases and controls by gestational age is highlighted in Fig 1. The average gestational age for the enrolled stillbirths (cases) was 33.8 ±3.2 weeks while that of the live births (controls) was 36.3±3.6 weeks, (*p-value* 0.244). Of all stillbirths, 41 were fresh stillbirths (FSB) and 55 were macerated stillbirths (MSB). Nearly two thirds 61(63.5%) of the stillbirths were males while the females were 35(36.5%). The mean age of the participating mothers was 30 years with majority aged between 20 to 34 years (70.1%). Age distribution across comparison groups was different with cases being aged (>35years) than their counterparts, 36.5% versus 21.9 respectively. About 59% of the participants were from rural and there were no significant differences among the groups. In relation to maternal education, cases were more common among less-educated women 50% versus 33.3% control group, history of stillbirth 13.5% versus 6.3% control. About 66% of mothers had good adherence to the antenatal clinic at least 4 visits, and those stillbirths were more prevalent to those who had less antenatal care visits 25% versus 5.2% in control. Obstetrical conditions presented during pregnancy included antepartum haemorrhage (APH) 14.2% (17.7% among cases versus 12.5% control; preeclampsia 20.8% (37.5% among cases versus 12.5%, Diabetes 4.2% which was similar in both cases and control group; and the anaemia 31.6% (39.6% among cases versus 27.6% in control group.

### Socio-demographic characteristics

Among socio-demographic factors that were significantly associated with stillbirth included advanced maternal age 35 years and above [OR(95%CI): 1.97(1.15–3.39)], less educated and secondary education [OR(95%CI): 3.75(1.67–8.41) and 2.35(1.04–5.28) respectively]. Some socio-demographic factors with positive association to stillbirth but did not show statistical significant were residing in rural [OR(95%CI): 1.02(0.62–1.68)], being employed [OR(95%CI): 1.05(0.63–1.73)], alcohol use [OR(95%CI): 1.57(0.71–3.47)] (Table 1). In other hands, adolescent mothers aged below 20 years and unmarried were negatively associated with stillbirth [OR(95%CI): 0.30(0.04–2.42)] and [OR(95%CI): 0.49(0.22–1.11)] respectively, (Table 1).

**Table 1:** Socio-demographic characteristics of the pregnant women seeking obstetrics care at KCMC, in Northern Tanzania, (n = 288).

Characteristics	Control (n = 192) n (%)	Case (n = 96) n (%)	OR(95%CI)
**Age, years**			
15–19	8(4.2)	1(1.0)	0.30(0.04–2.42)
20–34	142(74.0)	60(62.5)	1.00
≥35	42(21.8)	35(36.5)	1.97(1.15–3.39)
**Residence**			
Urban	79(41.2)	39(40.6)	1.00
Rural	113(58.8)	57(59.4)	1.02(0.62–1.68)
**Education**			
Primary/below	64(33.3)	48(50)	3.75(1.67–8.41)
Secondary	83(43.3)	39(40.6)	2.35(1.04–5.28)
College/above	45(23.4)	9(9.4)	1.00
**Marital status**			
Married	162(84.4)	88(91.7)	1.00
Single	30(15.6)	8(8.3)	0.49(0.22–1.11)
**Employment**			
Unemployed	120(62.5)	59(61.5)	1.00
Employed	72(37.5)	37(38.5)	1.05(0.63–1.73)
**Alcohol**			
No	176(91.7)	84(87.5)	1.00
Yes	16(8.3)	12(12.5)	1.57(0.71–3.47)

### Obstetrics characteristics

Among obstetrics factors, history of stillbirth was 2.34 times higher odds of developing stillbirth in the current pregnancy and this was statistically significant [OR(95%CI): 2.34(1.02–537)]. Mothers who did not attend or with one visit to ANC during pregnancy had 6.96 time higher odds of developing stillbirth when compared to those with at least four visits and this was statistically significant, [OR(95%CI): 6.96(3.11–15.57). Antepartum haemorrhage was significant factor for stillbirth [OR(95%CI): 2.54(1.27–5.08)] (Table 2). Obstetrics condition that showed a statistical significant association with stillbirth included preeclampsia [OR(95%CI): 4.19(2.32–7.62)]. Preterm premature rupture of membrane (PPROM) was positively associated with stillbirth but the association was not statistically significant [OR(95%CI): 2.103(0.81–5.49)], (Table 2).

**Table 2:** The obstetric characteristics of pregnancies resulting into stillbirths (n = 96) compared with live births (n = 192) among women seeking care at KCMC, Northern Tanzania.

Characteristics	Control (n = 192) n (%)	Case (n = 96) n (%)	OR(95%CI)
**History of stillbirth**			
No	180(93.8)	83(86.5)	1.00
Yes	12(6.2)	13(13.5)	2.34(1.02–537)
**Parity**			
Primiparous	88(45.8)	46(47.9)	1.00
Multiparous	84(43.8)	44(45.8)	1.01(0.60–1.67)
Grandmultiparous	20(10.4)	6(6.3)	0.57(0.22–1.53)
**ANC visits**			
≤1	10(5.2)	24(25.0)	6.96(3.11–15.57)
2–3	40(20.8)	23(24.0)	1.67(0.91–3.06)
≥4	142(74.0)	49(51.0)	1.00
**APH**			
No	174(90.6)	76(79.2)	1.00
Yes	18(9.4)	20(20.8)	2.54(1.27–5.08)
**Pre/eclampsia**			
No	168(87.5)	60(62.5)	1.00
Yes	24(12.5)	36(37.5)	4.19(2.32–7.62)
**PPROM**			
No	183(95.3)	87(90.6)	1.00
Yes	9(4.7)	9(9.4)	2.103(0.81–5.49)

APH: antepartum haemorrhage

PPROM: Preterm premature rupture of membrane

### Medical conditions

Among the medical conditions that showed a statistical significant association with stillbirth included anaemia [OR(95%CI): 1.72(1.02–2.88)]. Maternal obesity was positively associated with stillbirth but the association was not statistically significant [OR(95%CI): 1.74(0.91–3.35), (Table 3). Of all study participants, only 20(7%) were confirmed to be HIV-infected. Of these, 12(6.3%) were from the control group while 8(8.3%) were from the cases group. Similarly, out of all participants (N = 288), only 4 (1.4%) tested positive for malaria parasite infection; of these 3 were from a control group while the remaining was from the case group. Both HIV and malaria infections had no statistical association with stillbirth (Table 3).

**Table 3:** Medical conditions associated with stillbirths of women seeking care at KCMC, Northern Tanzania, (n = 288).

Characteristics	Control (n = 192) n (%)	Case (n = 96) n (%)	OR(95%CI)
**BMI group**			
18.5–24.9	72(37.5)	31(32.3)	1.00
25.0–29.9	84(43.8)	38(39.6)	1.05(0.59–1.86)
≥30	36(18.7)	27(28.1)	1.74(0.91–3.35)
**Diabetes in pregnancy**			
No	184(95.8)	92(95.8)	1.00
Yes	8(4.2)	4(4.2)	1.63(0.29–3.41)
**Anaemia**			
No	139(72.4)	58(60.4)	1.00
Yes	53(27.6)	38(39.6)	1.72(1.02–2.88)
**HIV sero-status**			
Yes	12(6.3)	8(8.3)	1.00
No	180(93.7)	88(91.7)	4 (3.4–5.7)
**Malaria infection**			
No	189(98.4)	95(99)	1.9(0.76–3.6)
Yes	3(1.6)	1(1)	1.00

BMI: Body mass index

### Multivariable characteristics

In multivariate logistic regression variables which remained statistically significant associated with stillbirth were low education level (primary education/below), [aOR(95%CI): 5.22(2.01–13.58)], history of stillbirth [aOR(95%CI): 3.17(1.20–8.36)], less antenatal visits 0 or 1 visits [aOR(95%CI): 6.68(2.71–16.48)], Preeclampsia [aOR(95%CI): 4.06(2.03–8.13)], and APH [aOR(95%CI): 2.39(1.04–5.53)], (Table 4).

**Table 4:** Multivariable logistic regression for odds ratio of stillbirth by associated maternal factors (n = 288).

Variable	N	Cases (n = 96) n(%)	aOR	95%CI
**Age, years**				
15–19	9	1(1.0)	0.17	0.02–1.53
20–34	202	60(62.5)	1.00	
≥35	77	35(36.5)	1.48	0.79–2.76
**Education**				
Primary/below	112	48(50.0)	5.22	2.01–13.58
Secondary	122	39(40.6)	4.28	1.62–11.26
College/above	54	9(9.4)	1.00	
**History stillbirth**				
No	263	83(86.5)	1.00	
Yes	25	13(13.5)	3.17	1.20–8.36
**Number of ANC visits**				
≤1	34	24(25)	6.68	2.71–16.48
2–3	63	23(24.0)	1.56	0.77–3.13
≥4	191	49(51.0)	1.00	
**Pre/eclampsia**				
No	228	60(62.5)	1.00	
Yes	60	36(37.5)	4.06	2.03–8.13
**Anaemia**				
No	197	58(60.4)	1.00	
Yes	91	38(39.6)	1.73	0.93–3.19
**APH**				
No	250	76(79.2)	1.00	
Yes	38	20(20.8)	2.39	1.04–5.53

ANC: Antenatal care clinics

APH: Antepartum haemorrhage

aOR: adjusted Odds Ratio

### Placenta pathologies

**Fig 2:**
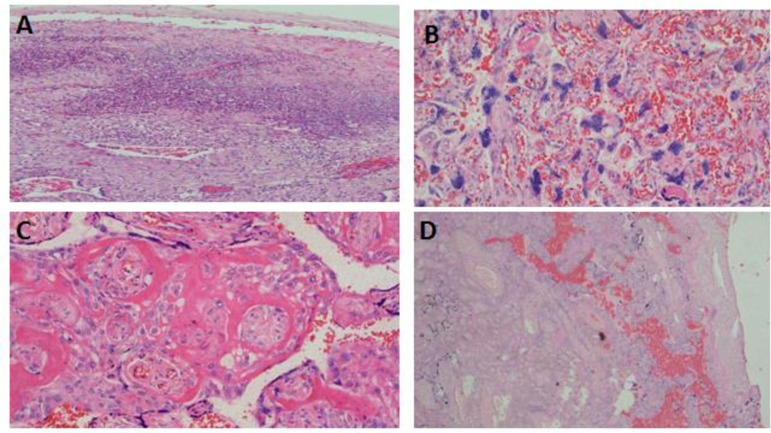
Histopathology of selected placenta pathological lesions among stillbirths at KCMC. Photomicroscopy demonstrating histopathology of various placental pathologies; presence of dense neutrophilic inflammatory infiltrates suggesting acute chorioamnionitis, H& E x 40 (A), prominent syncytial knots indicating uteroplancental vascular insufficiencies, H&E x 200 (B), perivillous fibrin deposition, H&E x200 (C), and infarction, fibrinoid necrosis causing collapse of chorionic villi and loss of cellular structures due to pyknosis, H&E x 63 (D).

**Fig 3:**
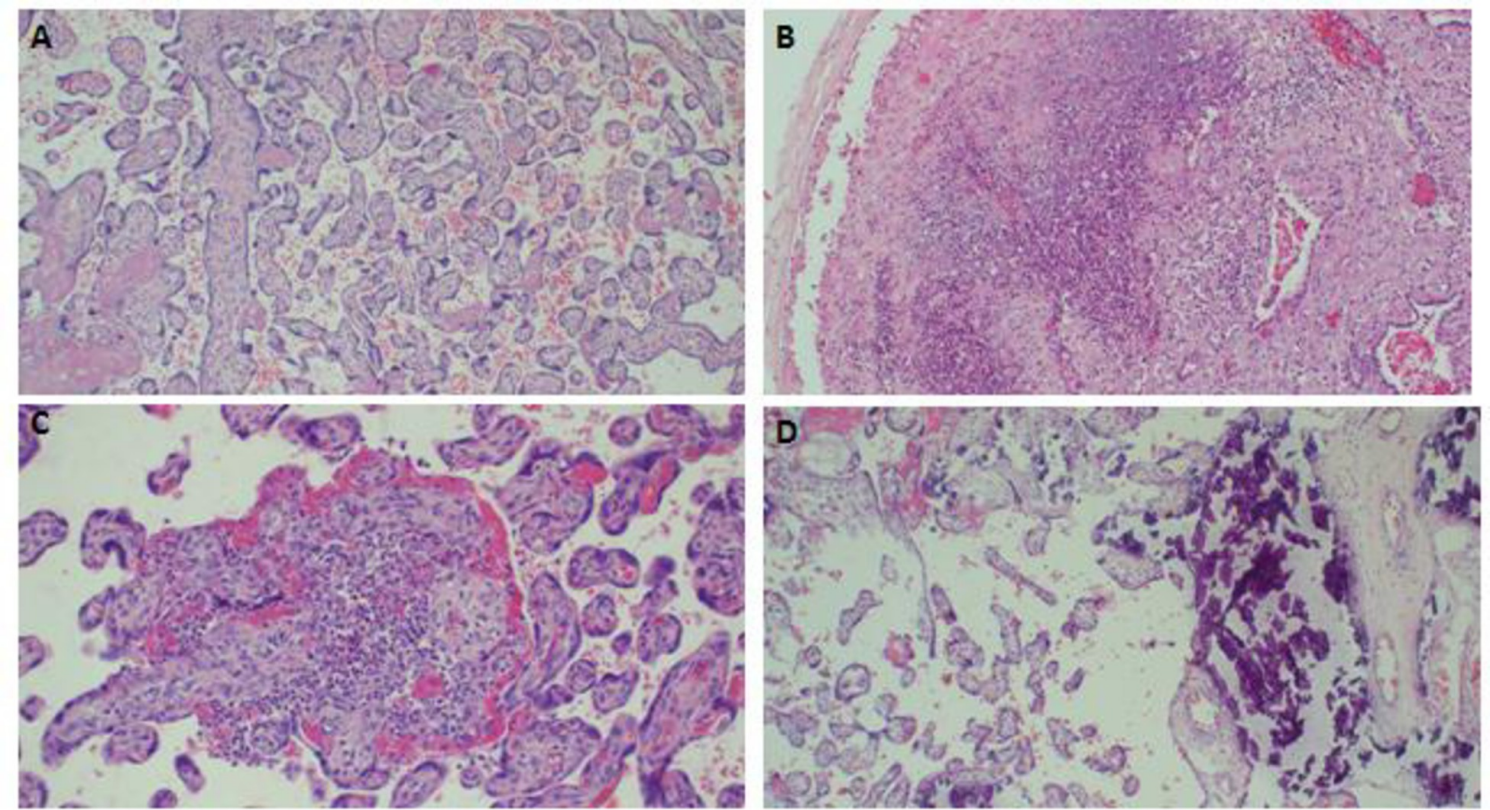
Histopathology of selected placenta pathological lesions among the control group (live births) at KCMC. Photomicroscopy of the placenta demonstrating non-significant pathology, H&E x 100 (A); diffuse neutrophilic inflammatory infiltrates compatible with acute chorioamnionitis, H& E x 40 (B), focal micro abscess compatible with placenta villitis, H&E x 200 (C), and presence of severe calcifications, H&E x 100, (D).

Placenta pathologies presented were uteroplacenta vascular pathology, acute chorioamnionitis, chronic inflammations, coagulation related lesion, and cord oedema. Histopathology of selected few placenta pathological lesions encountered among the cases and control groups are demonstrated in Figs 2 and 3 respectively.

The placenta pathology was analysed in a logistic regression model to determine their association with stillbirth. All placental pathology indicated positive association with stillbirth however, only three were statistically significant. These includes utero-placental vascular pathology [OR(95%CI): 13.19(7.33–23.75)], acute chorioamnionitis [OR(95%CI): 4.27(1.25–14.57), coagulation related lesion [OR(95%CI): 4.86(1.46–16.22)]. After adjusting odds ratios by some pathological features, the placental pathology that remained statistically significantly associated with stillbirth included uteroplacental vascular pathology [OR(95%CI): 13.06(6.33–26.97)] and acute chorioamnionitis [aOR(95%CI): [4.69(1.35–16.34)] (Table 5).

**Table 5:** Placental pathology associated with a stillbirth (n = 288).

Placenta pathology	Control (n = 192)	Cases (n = 96)	Unadjusted OR(95%CI)	Adjusted OR(95%CI)
Utero-placenta vascular pathology	34(17.)	73(76)	13.19(7.33–23.75)	13.06(6.33–26.97)
Acute chorioamnionitis	4(2.1)	8(8.3)	4.27(1.25–14.57)	4.69(1.35–16.34)
Chronic Inflammations	5(2.6)	5(5.2)	2.05(0.58–7.28)	2.25(0.63–8.03)
Coagulation related lesion	4(2.1)	9(9.4)	4.86(1.46–16.22)	1.06(0.30–3.79)
Cord oedema	1(0.5)	1(1.0)	2.01(0.12–32.50)	0.48(0.03–8.03)
Missing (normal placenta)	144(75)	-		

## Discussion

The current study has found that maternal education level, positive history of stillbirths, number of antenatal clinic visits, pre/eclampsia, anaemia, and ante partum haemorrhage were significant contributors to stillbirths. Similarly, utero-placenta vascular pathology and acute chorioamnionitis were placenta pathological findings that associated with stillbirths.

Maternal age is noted to be a risk of stillbirth in a number of studies, however, in the present study age was not a factor. Our findings are in contrast to the study done in UK, which revealed that advanced maternal age was a factor for stillbirth [8]. Similarly, the study conducted in Nigeria highlighted that advanced maternal age had the risk of developing stillbirths [9]. In line to other studies in Tanzania, Ghana and Canada; our study did not find a significant association between maternal age and risk of stillbirths [11, 13, 14]. The findings may suggest that the association between advanced maternal age and stillbirth risk is possibly independent of other known factors that could increase with age. This could be further explained by the fact that the likelihood of having hypertensive heart diseases, placenta pathologies and increased incidence of genetic and chromosomal abnormalities in women increase with advanced maternal age, thus a increasing risk of stillbirths [15].

The current study found strong association of maternal education and number of ANC attendance with an increased risk of stillbirth. Maternal education and counselling play important roles in the understanding of pregnancy warning associated with adverse outcomes such as stillbirth, thus the importance of antenatal care. Likewise, a research carried out in Nepal and Ethiopia reported that less-educated women and decresed antenatal visits were more likely resulted into stillbirths [16, 17].

The history of stillbirth increased the risk of stillbirth in the present study. Similar findings have been reported by a Cameroonian study which found that history of stillbirth was the most significant risk factor for stillbirths [18]. A systematic review study conducted in the UK also found that a history of stillbirth increased risk of recurrence rates [19]. The observed strong correlation between the positive history of stillbirths and high recurrences highlights a need for a close follow-up in of these vulnerable women in the subsequent pregnancies. Among obstetrics conditions, preeclampsia was found to have statistical evidence on stillbirth. The finding is supported by the previous study in the same setting using birth registry which also reported that preeclampsia was the main factor for stillbirth [20]. The findings agree well with the studies in developed counties which also reported that preeclampsia as the major risk of stillbirth. A systematic review in China reported that preeclampsia was a strong risk of stillbirth [21]. The risk of preeclampsia for stillbirth could be explained by the utero-placental vasculopathy frequently encountered in these cases [7]. However, our findings are contrary to the study in Cameroon which highlighted no association between preeclampsia and stillbirth. The differences could be due to difference in the data sources. The Cameroonian study used hospital records with limited maternal morbidity information particularly on obstetric history [18].

The current study found that placenta pathologies were much common in stillbirth compared to live births and the common lesions encountered were uteroplacental vascular pathology and chorioamnionitis. Reports on the association between placental pathology and stillbirth are not new. There is increasing evidence showing that placental pathologies may be the cause of stillbirth in pregnancies at gestation of 24 week or more [22–30].

Cord abnormalities such as insertion site, coiling index, implantation site abnormalities such as placenta previa, placenta accreta, increta and percreta; infectious disease processes (maternal or fetal) may compromise the circulation (maternal or fetal) [25–30]. Some of the conditions can be readily assessed by the obstetrician or midwife; however, others may require detailed histopathological assessment. Histopathology of the placenta in these lesions indicates acute and severe placental insufficiency, thereby causing stillbirth [22]. The current study results agree with a systematic reviews conducted in the UK by Ptacek and associates who reported a number of placental lesions to be associated with stillbirth; these included fetal thrombotic vasculopathy, endovasculitis, cord abnormalities, and delayed villous maturation. They also found inflammatory abnormalities such as choriomanionitis, villitis and endovasculitis also to be associated with stillbirth. Significantly, the researchers noted a wide spectrum of placental pathology and placental abnormalities in classification systems for identifying the cause of death in stillbirths. They highlighted the need to develop international consensus for diagnostic criteria and terminology to describe placental abnormalities in stillbirth [22].

Our study also agrees with another report in the USA which highlighted acute chorioamnionitis and other inflammatory lesions to be common in stillbirth and early preterm delivery [24]. This could be explained by an increase in infections in the placenta that is linked with causal pathology that precipitates labour leading to stillbirth and preterm deliveries. The placenta could be directly infected, leading into reduced blood flow to the foetus. Moreover, maternal infections of the genital tract or elsewhere may precipitate the preterm labour that foetus is unable to tolerate as the result of stillbirth [24].

This study had a number of limitations. Firstly, exclusion criteria were based on macroscopic assessment of congenital malformations of the stillbirths. Genetic or molecular tests for conditions such as Down's syndrome and others were not performed thus among the stillbirths included into this study, some might had genetic syndromes and thus influencing our placental pathology analysis. Secondly, limited data were available on microbiologic tests for the detecting infections in pregnant women diagnosed frequenty using TORCH panel which potentially may lead to significant changes in the placenta.

## Conclusions

Our study has demonstrated that uteroplacental vascular pathology and chorioamnionitis were the most placenta pathology lesions associated with stillbirth. Of the maternal medical conditions and obstetric characteristics that were assessed, less antenatal visits, preeclampsia, and history of stillbirths were the major risk factors associated with stillbirth. Consistent antenatal clinic attendance is recommended since it provides an opportunity to screen for potential risk factors for stillbirth as well as counselling to women thus ensuring successful pregnancy outcome. Histopathological examination of placentas of unexplained stillbirth deliveries is recommended as this knowledge may help in preventive measures which would lower the rates of stillbirth deliveries. Examination of the placenta may help to identify causation and indicate treatment options in subsequent pregnancies. The findings in the current study may stimulate interest among obstetricians and pathologists to investigate the placenta wherever possible in the stillbirth population that would help provide information about stillbirth etiology. Further studies are needed on determinants of stillbirth such as those on post mortem statistics, genetic and molecular testing, microbiology, foetal blood/urine/ culture to better characterize the specific causes of stillbirth.

## Definition of terms

1. **Early preterm:** A baby born with gestation age between 28 +0 day to 33 weeks +6 days, [31, 32].
2. **Late preterm:** A baby born between 34 weeks +0/7 days and 36weeks +6/7 days [31, 32]
3. **Term delivery:** Refers to baby born after 37 weeks +0 day gestation up to 41 weeks [33, 34]
4. **Preterm premature of membrane (PPROM):** Refers to rupture of fetal membrane prior to 37 weeks of complete gestation [35]
5. **Antepartum hemorrhage (APH):** Refers to bleeding from or in to the genital tract occurring after 28weeks gestation and prior to birth of the baby [36]
6. **Fresh stillbirth:** Is the baby born dead without sign of skin disintegration, and death is assumed to occur less than 12 hours prior to delivery [31]
7. **Macerated stillbirth:** Refers to baby born dead with signs of skin and soft tissue disintegration, and death is assumed to occur more than 24 hours prior to delivery [31].

## Supporting information

S1 Questionnaire (PDF)
