# Supplementary material for: Placental pathology and maternal factors associated with stillbirth: An institutional based case-control study in Northern Tanzania
Source: PLoS One. 2020 Dec 31;15(12):e0243455. doi: 10.1371/journal.pone.0243455 (PMC7775101; doi:10.1371/journal.pone.0243455)
Supplement: S1 Questionnaire — (PDF) [file pone.0243455.s001.pdf]

K.C.M.C

KILIMANJARO CHRISTIAN MEDICAL CENTRE

An institution of the Good Samaritan Foundation

P.O. Box 3010, Moshi, Tanzania

Tel: 255-027-2754377 / 80 Fax: 255-027-2754381

Email: kcmcadmin@kcmc.ac.tz Website: http: / /www.kcmc.ac.tz

QUESTIONNAIRE

STUDY ID No……………………………Date of enrollment……..…………………………………….

PLACENTAL PATHOLOGY AND MATERNAL FACTORS ASSOCIATED WITH STILLBIRTH AMONG DELVERIES AT KILIMANJARO CHRISTIAN MEDICAL CENTRE FROM OCTOBER 2018 TO MAY 2019

PART A

1. Maternal age (years)………………………….
2. Address/Residence……………………………………………..
3. Religion……………………………………
4. Tribe……………………………………………..
5. Weight (kg)……………. Height (m)…………………… BMI (kg/m2)……………………
6. BP (mmHg)………….
7. RBG……………………
8. HB……………………..
9. Blood group…………… RH…………….
10. If negative, any previous ant D immunization.. 1 yes 2. no
11. Education level.
    1. No formal education
    2. Primary
    3. Secondary
    4. College/university

12. Marital status
    I. Single
    II. Married
13. Employment status
    I. Not employed
    II. Employed

14. Tobacco use 1. Yes 2. No
15. Alcohol use 1. Yes 2. no
16. Comorbidity of the mother
    1. Chronic Hypertension
    2. Severe pre eclampsia/eclampsia
    3. Heart disease
    4. Diabetes mellitus

5. HIV/AIDS
6. Previous scars
7. PPROM/PROM
8. Others. Specify………………..

17. Previous stillbirth…. 1.yes. 2.no
18. Previous abortion…… 1. Induced. 2. Spontaneous
19. Placenta location
    1. normal
    2. Previa
    3. abruptio
20. Gravidity………………… parity……………… Gestation Age (at delivery)…………………….
21. Antenatal visits…………………..
22. Mode of delivery: 1. SVD. 2.C/S
23. Ante partum hemorrhage ……………………
24. Hb level after delivery………………………………………….
25. Gross examination of the placenta and membrane……………….…………………
    Dimensions……………………….
    Weight of placenta……….gm
    Length of the cord…………cm
    Overt presence of infection……………………., Ischemia……………………..Necrosis

26. Cord length…………………………..
27. Cord appearance……………………………….
    1. Insertion.. a. normal, b. marginal
    2. Cord knot
    3. Velamentous cord
    Others, Specify……………………………
28. Cord location…. 1.normal. 2.Nuchal cord. 3. Cord prolapsed. 4. Cord presentation
29. State of the baby
    1. Live birth
    2. Stillbirth

27. If stillbirth: 1. FSB 2. MSB

28. Birth weight………… (Zhang and Watson curve)……………………..
    1. SGA
    2. NORMAL
    3. LGA

29. Fetal appearance 1. Normal 2. Abnormal… specify………..

PART B:

PATHOLOGY ANALYSIS OF THE PLACENTA SPECIMEN

II. PRE-ANALYTICAL ASSESSEMENT

1. Date and time the placenta sample was received in the laboratory…………………….
2. Sample received by……………………………………………………………………….
3. Was the sample well labeled……………………………………………
4. Fixative used……………………….adequacy……………………………….
5. Name of the person who performed fixation……………………….
6. Time when fixation ended…………………………………………….
7. Fixation duration……………………………………………………..
8. Any comments about fixation…………………………………………
9. pH of the formalin used when it was prepared……………………………….
10. Date formalin prepared………………………………………………..
11. Time the sample was placed in the formalin…………………..
12. Grossing date and time……………………
13. Grossing performed by (name of the person)………………..
14. Number of cassettes…………………………..
15. Date of processing machine…………………..
16. Date of cuts……………………………………..
17. Number of slides cut……………………………
18. Date of staining……………………………..

III. MICROSCOPIC ANALYSIS OF THE PLACENTA AND MEMBERANCES

1. Specimen quality…………Excellent………Good…………Satisfactory………..Poor………
2. Ischemic changes………………
3. Autolysis………………………..
4. Necrosis…………………………..
5. Acute chorioamninitis………………………………
6. Chronic inflammation with features of villitis……………………………
7. Uteroplacental vascular pathology and evidence of secondary villous damage…………………
8. Coagulation related lesions in the presence of ureteroplacental vascular thrombosis………
9. Placenta chorioangiomas…………………………….
10. Normal histology……………………………

B: umbilical cord

1. Cord oedema………………………………….
2. Necrosis of the cord…………………………
3. Thrombosed cord……………………………….
4. Cord compressiontrue knot in cord……………….
5. Cord hematoma …………………………………
6. Cord torsion……………………………………
7. Cord congestion………………………………….
8. Number of vessels (a) 1 (b) 2). (c). >2……………..
9. Normal………………………………

C: Primary pathologic diagnosis classification

1. Placental factor
2. Cord complications

3. Fetal factors
4. Maternal factors
5. Unknown (with or without maternal factors)

Diagnosis/Report made by……………………………………………….
